# An *ex vivo* model for education and training of bilateral cleft lip surgery

**DOI:** 10.1186/s12909-023-04575-9

**Published:** 2023-08-18

**Authors:** Rainer Lutz, Marco Rainer Kesting, Manuel Weber, Manuel Olmos, Deniz Tasyürek, Tobias Möst, Jan Bürstner, Katja Leonie Schulz

**Affiliations:** Department of Oral and Cranio-Maxillofacial Surgery, University Hospital Erlangen, Friedrich-Alexander University Erlangen-Nürnberg (FAU), Glückstrasse 11, 91054 Erlangen, Germany

**Keywords:** Bilateral cleft lip, Lip plasty, Cheiloplasty, Porcine snout disc, *Ex vivo* model, Cadaver model, Surgical simulation, Residency training, Skills lab, Teaching

## Abstract

**Background:**

Bilateral cleft lip surgery is very challenging and requires a high level of skill, knowledge and experience. Existing high-fidelity simulation models that can be used by novice cleft surgeons to gain experience and expand their knowledge are rare and expensive. In this study, we developed a bilateral cleft lip model using porcine snout discs, which are available anywhere and inexpensive.

**Methods:**

Anatomic reference points of a patient with a bilateral cleft lip were superimposed with landmarks of the porcine snout disc on a foil template. The template was used to construct an *ex vivo* bilateral cleft lip model. Surgery was performed on the model according to Millard and the surgical steps were photodocumented analogous to two clinical cases of bilateral cleft lip surgery. The suitability of the model was further tested by twelve participants and evaluated using self-assessment questionnaires.

**Results:**

The bilateral cleft lip *ex vivo* model made of a porcine snout disc proved to be a suitable model with very low cost and ease of fabrication, as the template is reusable on any snout disc. The Millard procedure was successfully performed and the surgical steps of the lip plasty were simulated close to the clinical situation. Regarding the nasal reconstruction, the model lacks three-dimensionality. As a training model, it enhanced the participants comprehension of cleft surgery as well as their surgical skills. All participants rated the model as valuable for teaching and training.

**Conclusions:**

The porcine snout discs can be used as a useful *ex vivo* model for bilateral cleft lip surgery with limitations in the construction of the nose, which cannot be realistically performed with the model due to anatomical differences with humans. Benefits include a realistic tissue feel, the simulation of a multi-layered lip construction, a wide and rapid availability and low cost. This allows the model to be used by novice surgeons also in low-income countries. It is therefore useful as a training model for gaining experience, but also as a model for refining, testing and evaluating surgical techniques for bilateral lip plasty.

## Background

Treatment of bilateral cleft lip deformities is one of the most challenging procedures in cleft and craniofacial surgery [1]. Successful treatment can only be achieved if the surgeon has a thorough knowledge not only of the desired anatomical situation, but also of the pathological situation and the various ways to modify it in order to achieve an excellent postoperative result [1]. As opposed to many other fields of plastic and reconstructive surgery, bilateral cleft lip surgery is not a matter of replacing lost tissue. The surgical intervention rather aims to release the improperly attached and displaced anatomical structures and carefully realign them in the correct manner, taking into account postoperative growth [2]. Somewhat like solving a three-dimensional puzzle with varying degrees of difficulty in the fourth dimension. In detail this includes establishing a functional orbicularis oris muscle, creating a symmetric cupid’s bow and upper lip, as well as symmetric nostrils and improving the nasal support, while keeping secondary deformities to a minimum [1]. In addition to the difficulties of a unilateral cleft, surgeons have to deal with the absence of developed lip tissue in the prolabial segment as well as widely displaced premaxillary and lateral lip segments [3]. In 1952, Brown phrased very aptly that surgical repair of bilateral clefts was about twice the problem than of unilateral clefts, while the results were only about half as good [4]. Even later the aesthetic outcomes have predominantly been inferior to the outcomes of unilateral cleft surgery [5]. In the early years (15th − 17th centuries), the problem was radically solved e.g. by removing the premaxilla to facilitate lip closure. Damage, which has been caused to the natural facial growth process, however, cannot be undone and the surgical outcome will impact on many parts of the child’s life [5, 6]. This charges a great responsibility on the surgeons, who are obliged to fully comprehend the complexity of the surgery before conducting it on a patient [3]. Since the results of cleft lip surgery are highly dependent on the surgeon’s skill, knowledge and experience [7], ways to increase these very matters should be easily accessible. Surgical training models have already proven to be beneficial in reconstructive maxillofacial surgery [8–10]. As they allow surgeons to learn from errors without severe consequences and to gain skill, confidence and experience, they should be easily available and accessible as well as affordable [11, 12].

Simulation models for cleft surgery exist, but are – according to current literature research (02/2023) – mostly limited to the simulation of unilateral clefts. To our knowledge only Smile Train/Simulare Medical provides a bilateral cleft lip simulator (https://www.smiletrain.org/simulare/bilateral-simulator). However, we found no scientific evaluation of this model. Cleft lip simulators are classified as digital simulators and high- or low-fidelity haptic simulators [11]. Digital simulators are able to enhance one’s theoretical knowledge, but cannot increase practical skills [12]. Low-fidelity models, on the other hand, are capable of both, but only to a certain extent. They do not provide different tissues [11] and therefore no possibility of a multi-layered lip construction. Considering that restoring normal muscular function is a key factor for successful treatment [13], only high-fidelity models are eligible for achieving an adequate theoretical and practical learning experience. At the current time, however, high-fidelity models are rare even for unilateral cleft lip surgery, are associated with high costs and are solely synthetic-based [11, 12]. Yet the dimensional stability of the silicone models is much higher than of the human skin and optimal surgical results are hitherto impossible to achieve on silicone models [14]. The superiority of cadaver models in terms of realistic tissue handling is already outlined in comparative studies [15, 16]. In other fields of reconstructive oral and maxillofacial surgery, porcine models have already been successfully utilised [9, 10, 17]. The part of the pig’s body that resembles the human upper lip region is the porcine snout disc. It not only shows anatomical and histological similarities to the human region of interest, but is also in line with the 3R principle *(“replace, reduce, refine”; published by Russell an Burch* in 1959) for the reduction of animal experiments, since it is an *ex vivo* model and uses only parts of low culinary utilisation value [18–20]. Therefore, the aim of this study is to develop an *ex vivo* porcine snout disc model for simulation and training of bilateral cleft surgery, which provides a great learning experience, is as realistic as possible, widely available, easily accessible and cost effective.

## Methods

### Establishing an *ex vivo* model of a bilateral cleft

**Fig. 1:**
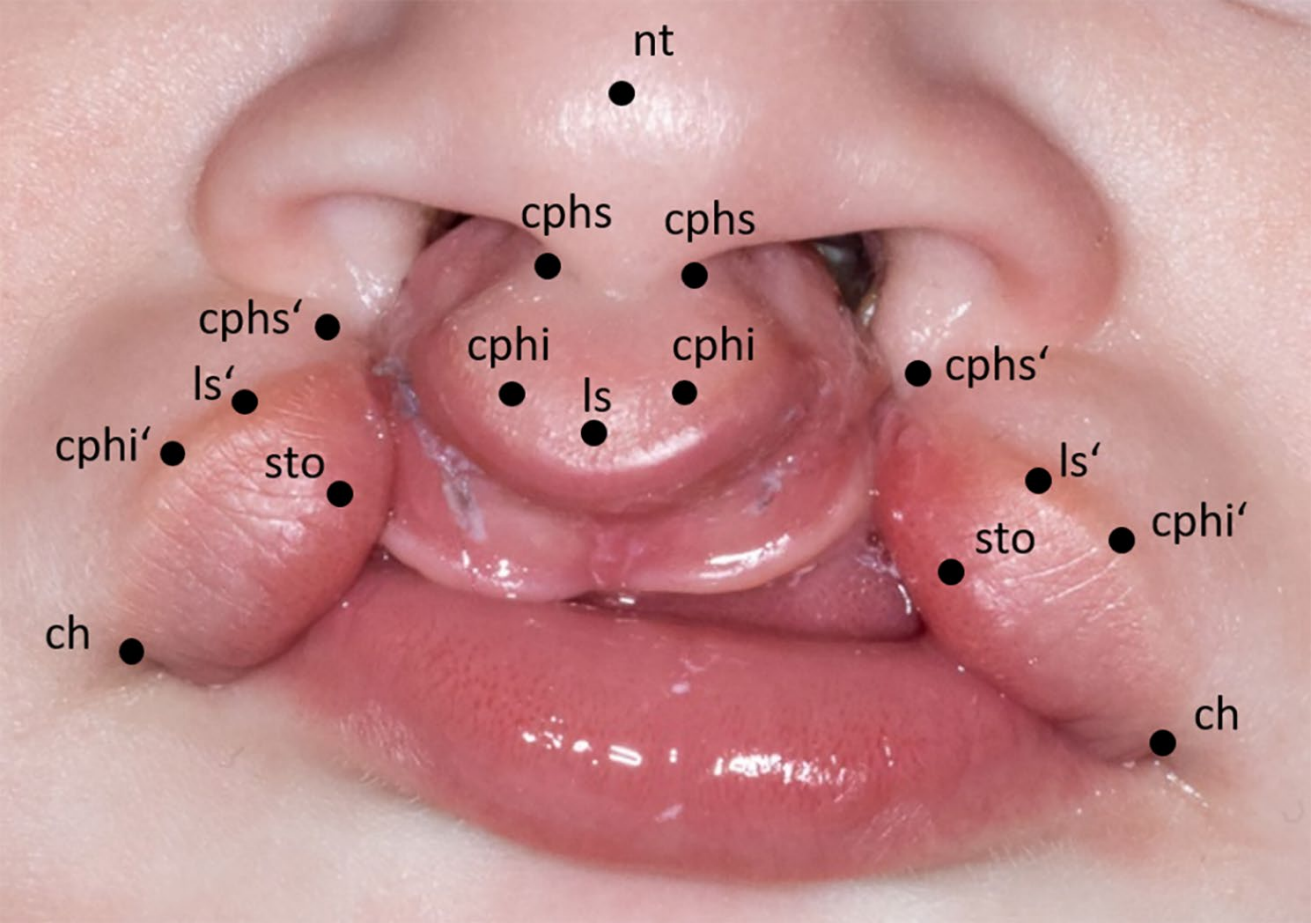
Anatomic reference points on a picture of a cleft patient with bilateral cleft lip and palate: nasal tip (nt): tip of nose; crista philtri superior (cphs): highest point of philtrum edge; crista philtri inferior (cphi): tip of the cupid’s bow; labiale superius (ls): median (lowest point) of cupid’s bow; stomion (sto): lowest median point of upper lip; cheilion (ch): lateral commissure of the lip

Porcine snout discs were provided by a local company (Contifleisch GmbH, Erlangen, Germany). The standard slaughter age of the pigs is six months. We stored the fresh snouts moist and cool until use to maintain optimal tissue texture. The uniformity of the porcine snout discs and the magnification scale have been determined prior to the study (data not shown in this study). The anatomical situation of a cleft patient with a bilateral cleft lip and palate served as a reference for designing the *ex vivo* model. A picture of the patient was printed in double magnification as to fit the porcine snout discs’ size and anatomic reference points were highlighted (Fig. 1).

**Fig. 2:**
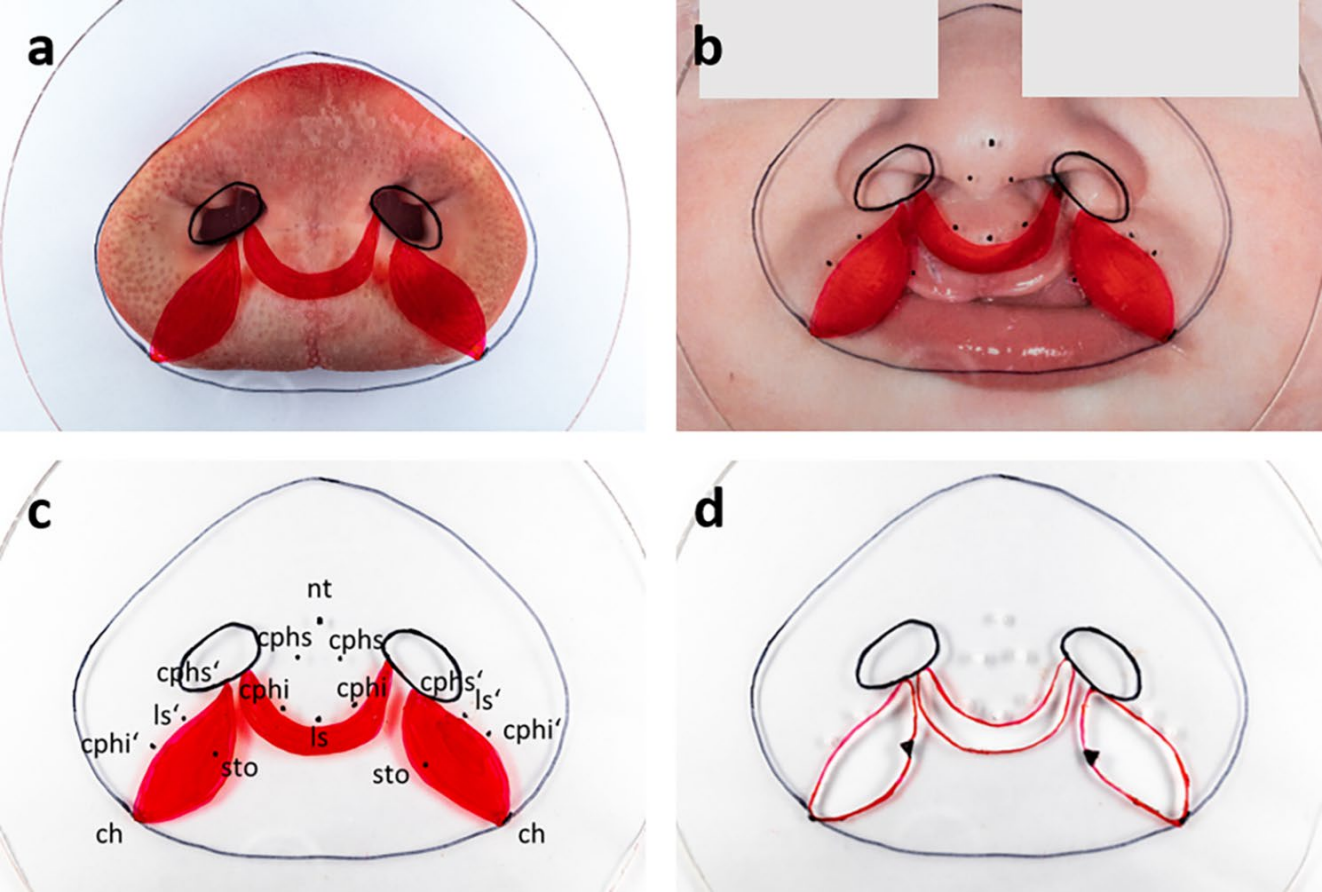
Template foil with anatomic landmarks of both snout disc and cleft child: on the porcine snout disc (**a**), on a double magnified picture of the cleft child with anatomic reference points (**b**), with labelled reference points (**c**), final template foil with drill holes (**d**)

On this basis, a template was created using a thermoforming foil made of polyethylene terephthalate (dimension 1.5 × 125 mm, Erkodur, Erkodent Erich Kopp GmbH, Germany) as shown in Fig. 2.

**Fig. 3:**
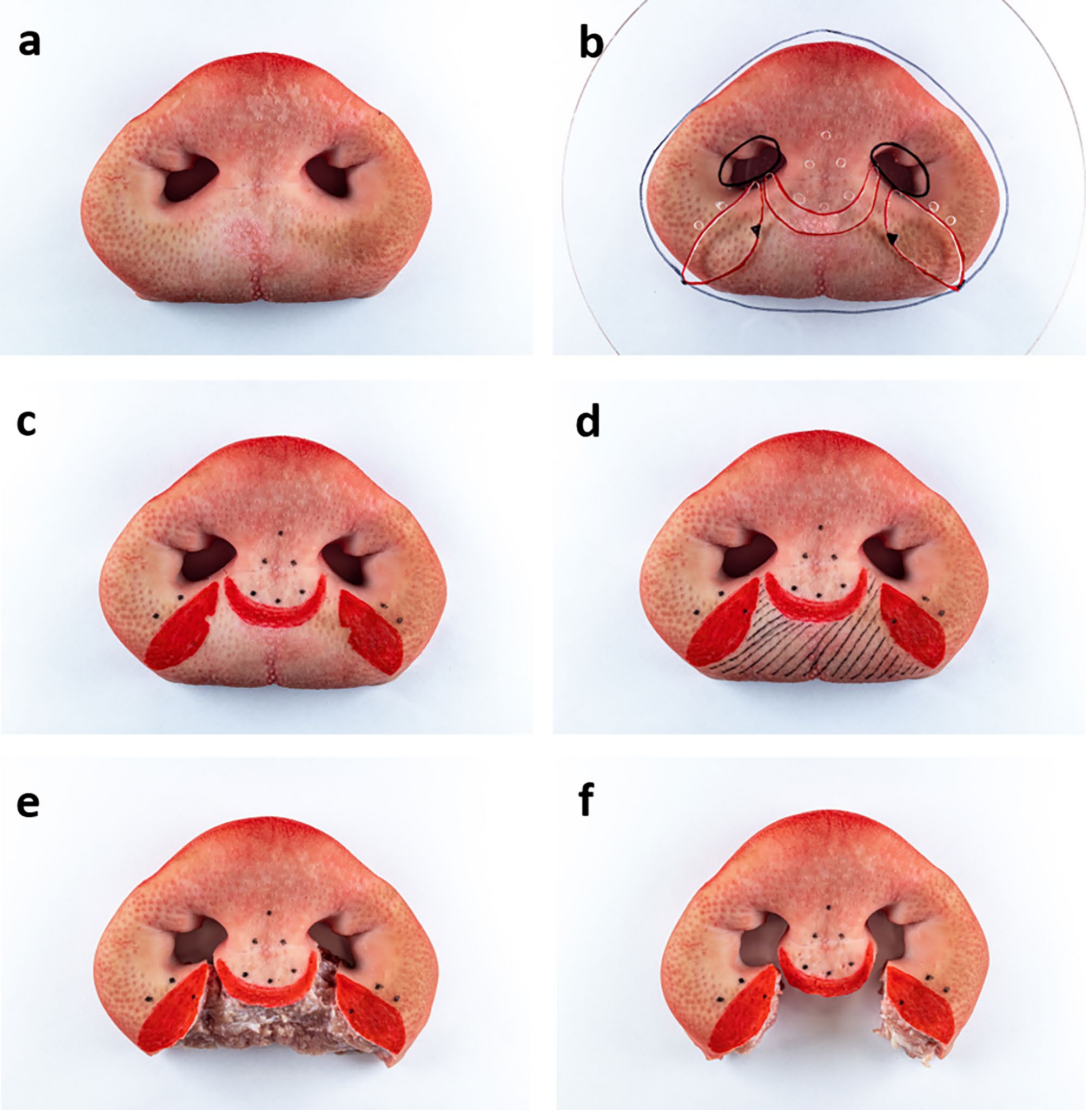
Creation of the bilateral cleft lip model: porcine snout disc (**a**), with template foil (**b**), after marking the anatomic reference points (**c**), with sto points marked and cleft area shaded (**d**), after skin excision (**e**), after muscle excision: final *ex vivo* model (**f**)

**Fig. 4:**
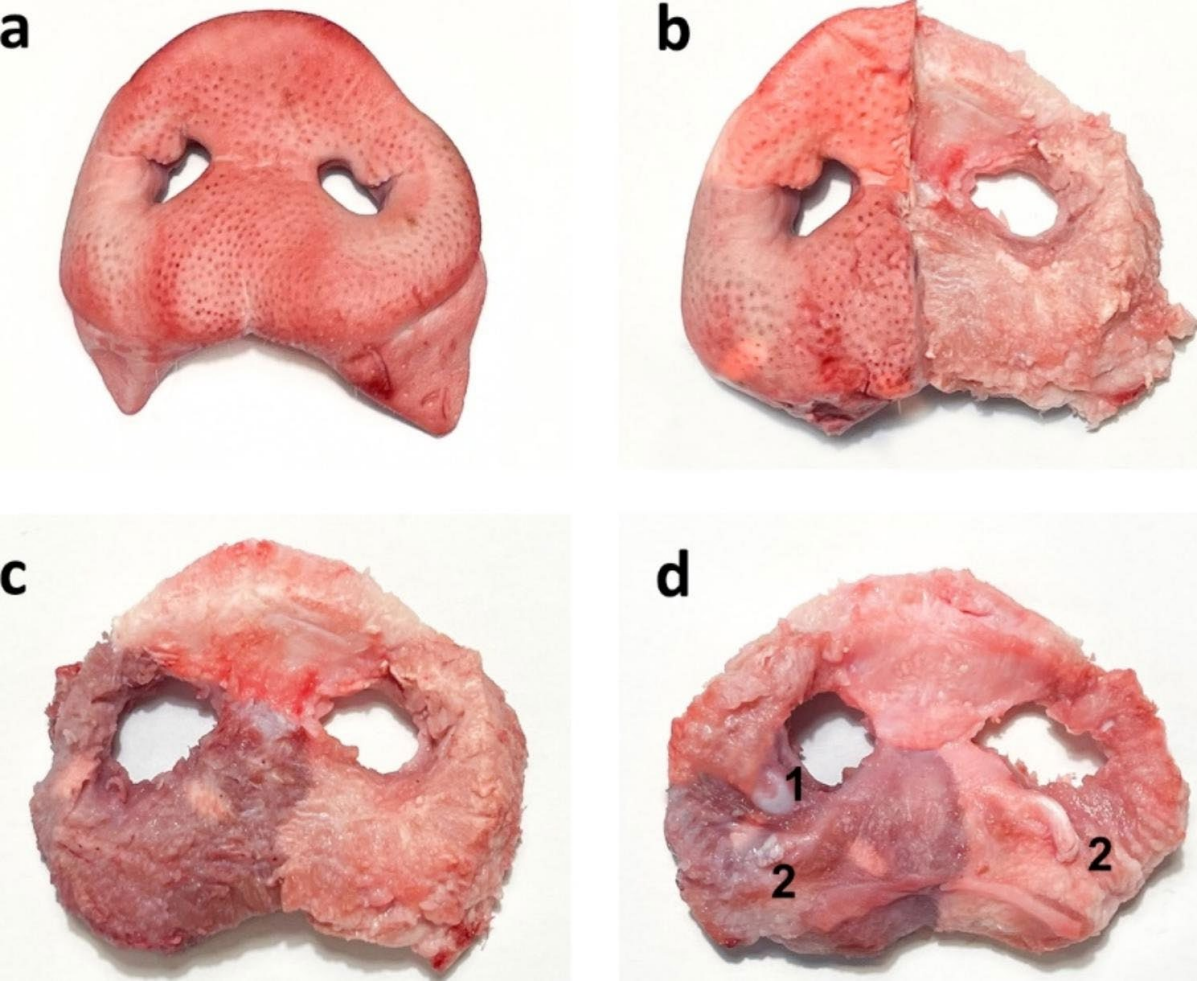
Anatomy of the porcine snout disc: snout disc before preparation (**a**), after the skin on the left half of the pig’s snout was removed to show the relationship between skin and muscle (**b**), after removal of the skin, visualizing the main component of the snout disc: M. nasalis, a muscle which has no attachment to the bony skeleton of the pig’s skull (**c**), view from the back of the nasal disc: the Cartilago nasi lateralis ventralis (1) and the end tendons of the caninus muscle (2) which is closely interwoven with the levator labii superioris muscle, radiate into the nasal muscle (**d**)

Anatomic landmarks and reference points of both the porcine snout disc and the cleft patient were marked on the foil template with foil markers (STAEDTLER non-permanent Lumocolor, STAEDTLER Mars GmbH & Co. KG, Nuremberg). After piercing points on the foil with a drill and cutting out the vermillion region except the markings for the sto points, the foil template was ready to use. By orientating on the anatomic landmarks, the foil was now placed on a porcine snout disc. Vermillion and anatomic reference points were marked through the holes in the foil onto the snout disc and the cleft area was shaded. The clinical cleft situation was now displayed on the snout disc. Then, the cleft area was cut and excised. The porcine nasal muscle, which is the internal muscle of the snout disc [21], was now prepared to resemble the human muscle stumps and the muscle of the prolabium was removed to create the final *ex vivo* model (Fig. 3). A detailed overview of the musculature of the snout disc is given by the prepared snout in Fig. 4. In addition to the skin and a piece of cartilage, e.g. Cartilago nasi lateralis ventralis (1 in Fig. 4d), the snout disc consists of the internal nasal muscle and muscle tendons, e.g. from M. caninus (2 in Fig. 4d), which radiate into the snout disc.

### Proof of concept –performing bilateral cleft surgery with the Millard technique on the *ex vivo* model

**Fig. 5:**
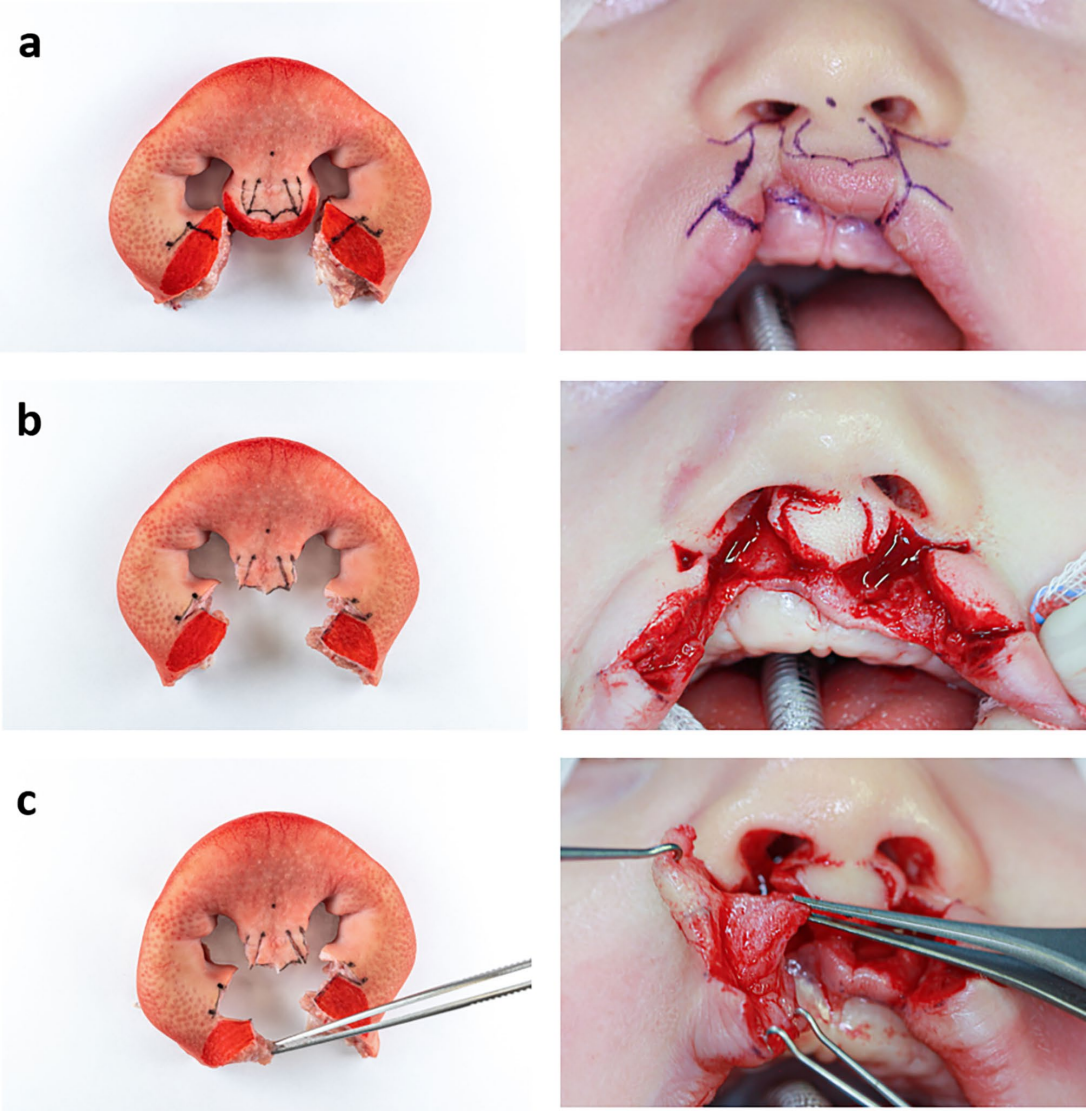
Millard surgery of a bilateral cleft on the *ex vivo* model (left) and patient A (right), part one: initial situation with drawn incision lines (**a**), after incisions and vermillion cut out (**b**), mobilised orbicularis oris muscle (**c**)

**Fig. 6:**
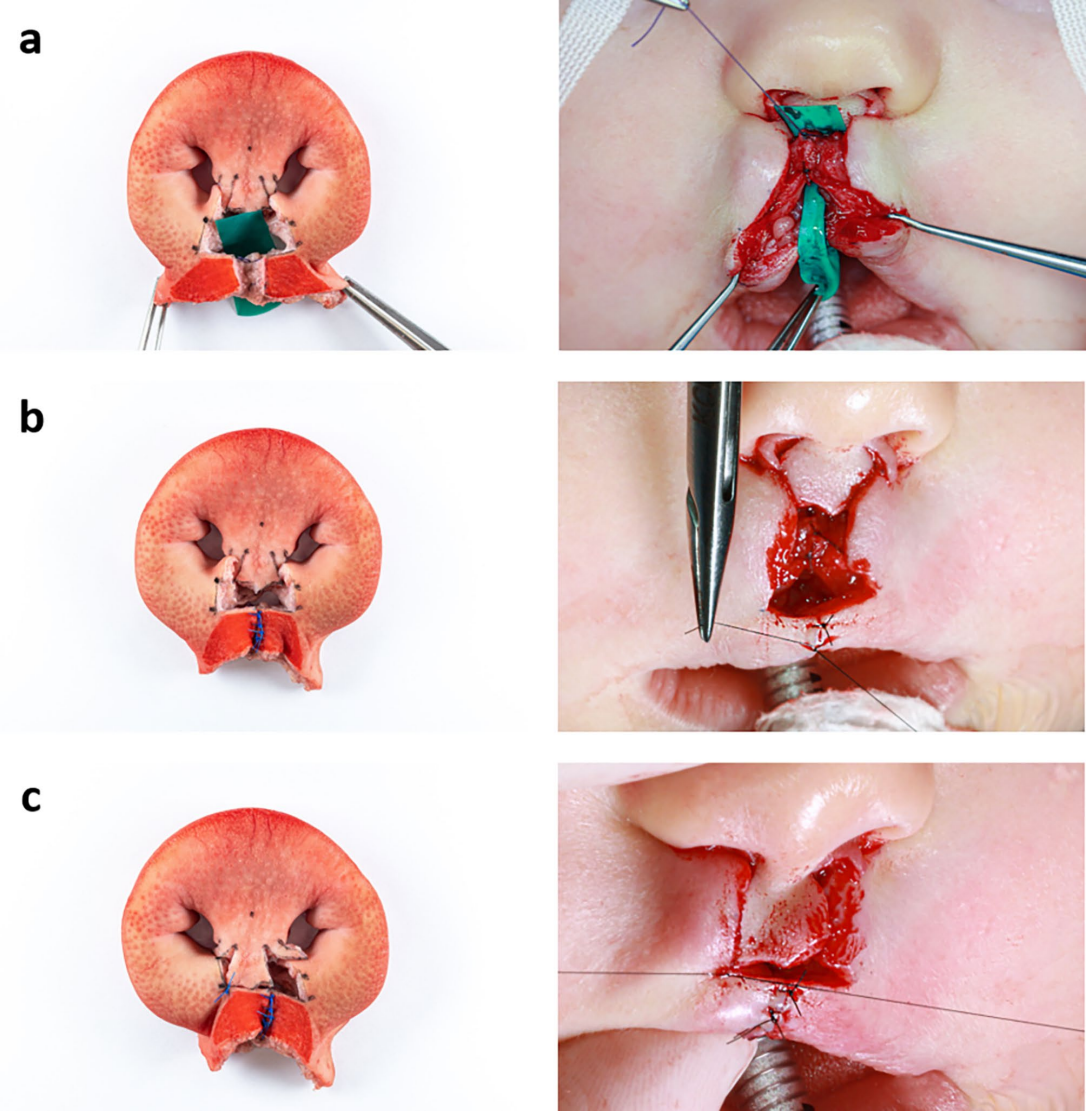
Millard surgery of a bilateral cleft on the *ex vivo* model (left) and patient A (right), part two: suture of the orbicularis oris muscle (**a**), suture of the vermillion (**b**), suture fixating the prolabium to the right lip segment on point cphi (**c**)

**Fig. 7:**
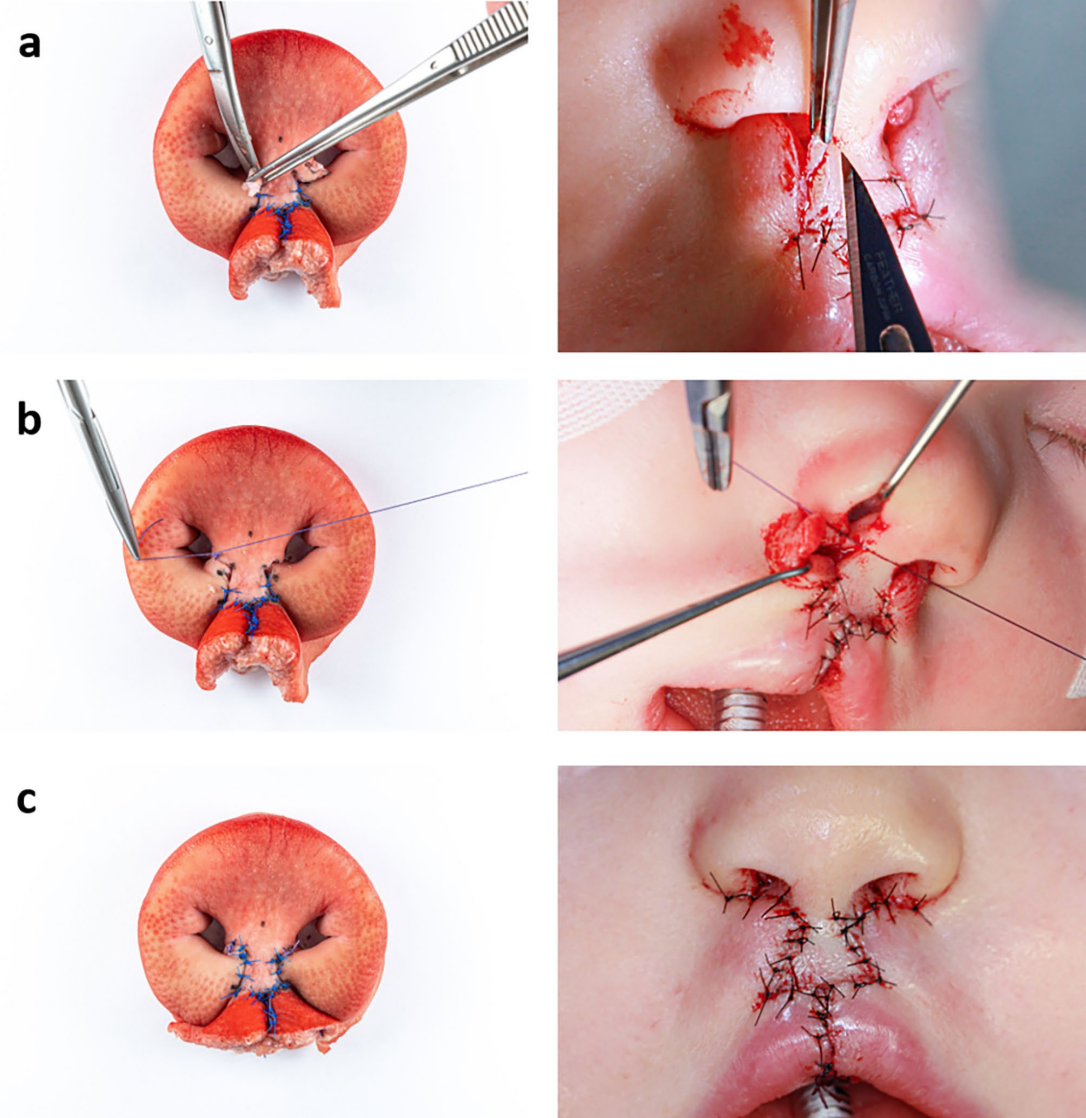
Millard surgery of a bilateral cleft on the *ex vivo* model (left) and patient A (right), part three: removing the parking flaps (**a**), suture of the nasal entrance (**b**), finished skin suture and final result (**c**)

**Fig. 8:**
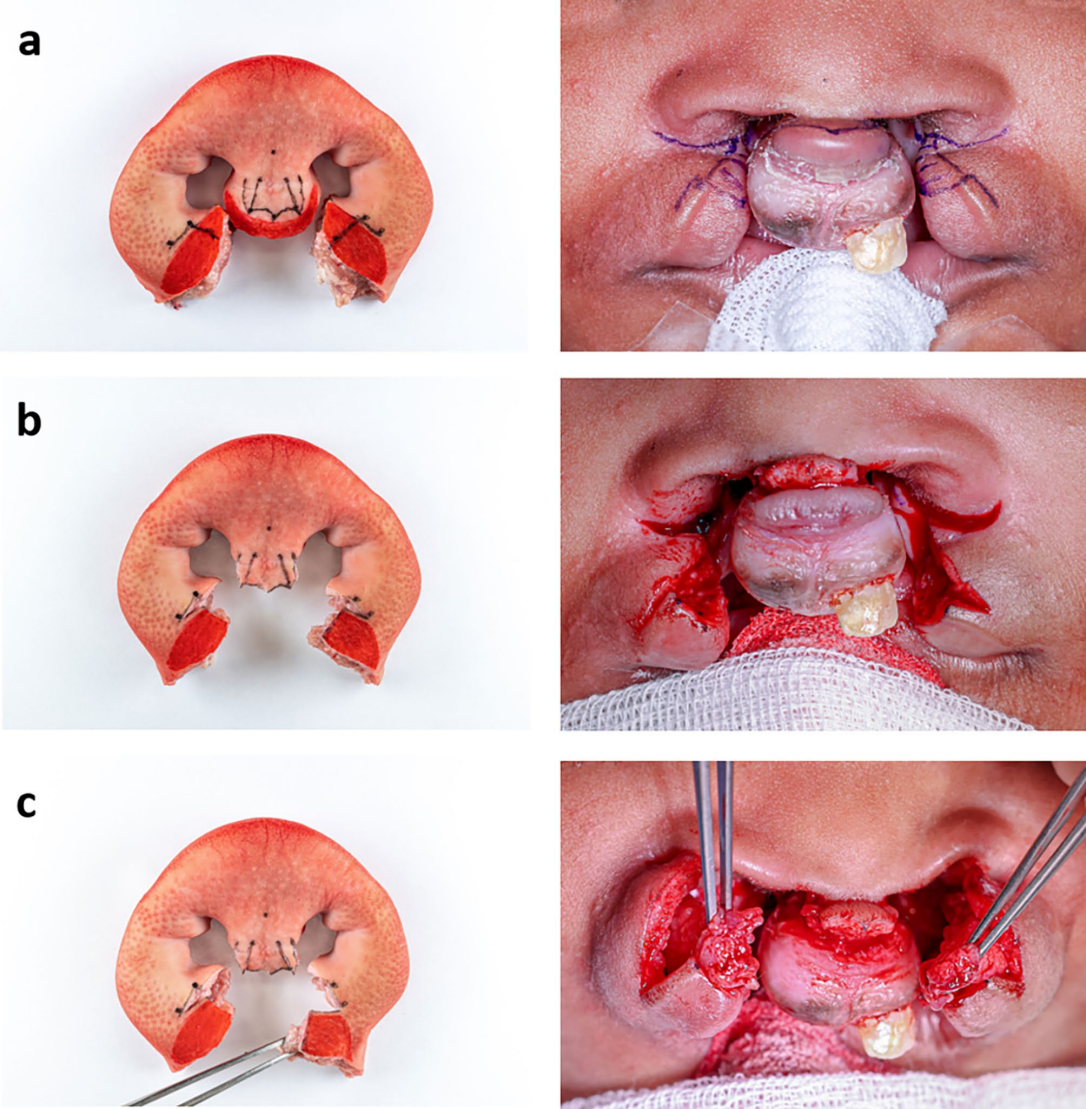
Millard surgery of a bilateral cleft on the *ex vivo* model (left) and patient B (right), part one: initial situation with drawn incision lines (**a**), after incisions and vermillion cut out (**b**), mobilised orbicularis oris muscle (**c**)

**Fig. 9:**
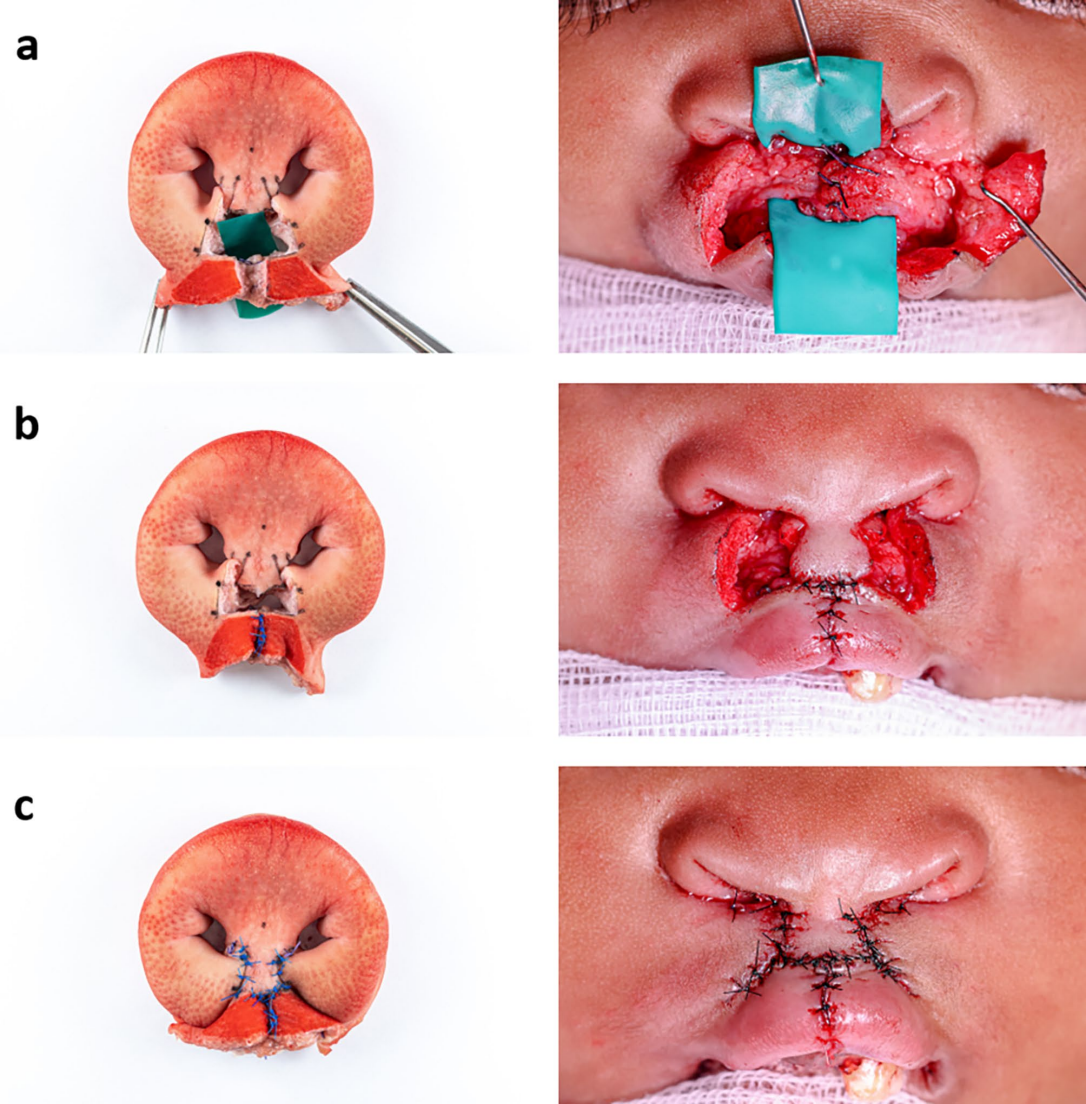
Millard surgery of a bilateral cleft on the *ex vivo* model (left) and patient B (right), part two: attached orbicularis oris muscle (**a**), suture of the vermilion, in the patient the prolabium has already been sutured to the vermillion (**b**), completed suture of the skin (**c**)

In order to validate the eligibility of the developed model, Millard technique was performed on it by an experienced surgeon. For a clear visualisation of the surgical procedure, the surgical steps of two patient cases were correlated with the newly created model (Figs. 5, 6, 7, 8 and 9) to show that the steps of bilateral cleft surgery can be reproduced on the *ex vivo* model.

First, incision lines were drawn in according to Millard (Figs. 5a and 8a).

Then, the incisions were made and the muscle-free cupid’s bow (cphi-ls-cphi) and the medial parts of the vermilion were removed. An incision was then made along the “white roll” up to the cphi’ points. The prolabium was prepared and the c-flaps were incised as “parking flaps” (Figs. 5b and 8b). If necessary, these can later be used to lengthen the columella by rotating them into the columellar base.

The next step was the further dissection of the orbicularis oris muscle (Figs. 5c and 8c).

After the model was completely prepared, cleft closure began with the suture of the orbicularis oris muscle with Vicryl 4 − 0 (Figs. 6a and 9a) and the precise adaptation of the cranial parts of the vermillion (ls’) with Ethilon 4 − 0 (Figs. 6b and 9b).

The vermillion was now adapted entirely. Next, the cphi points of the prolabium were fixed to the corresponding cphi’ points on both sides (Figs. 6c and 9b).

As they were not needed in this case, the parking flaps were removed (Fig. 7a). Next followed the construction of the nasal entrances (cphs) with Vicryl 4 − 0 (Fig. 7b). By completing the suture with Ethilon 4 − 0 in the area of the newly created cupid’s bow and the prolabium, the cleft closure was completed (Fig. 7c an 9c).

### Evaluation

**Fig. 10:**
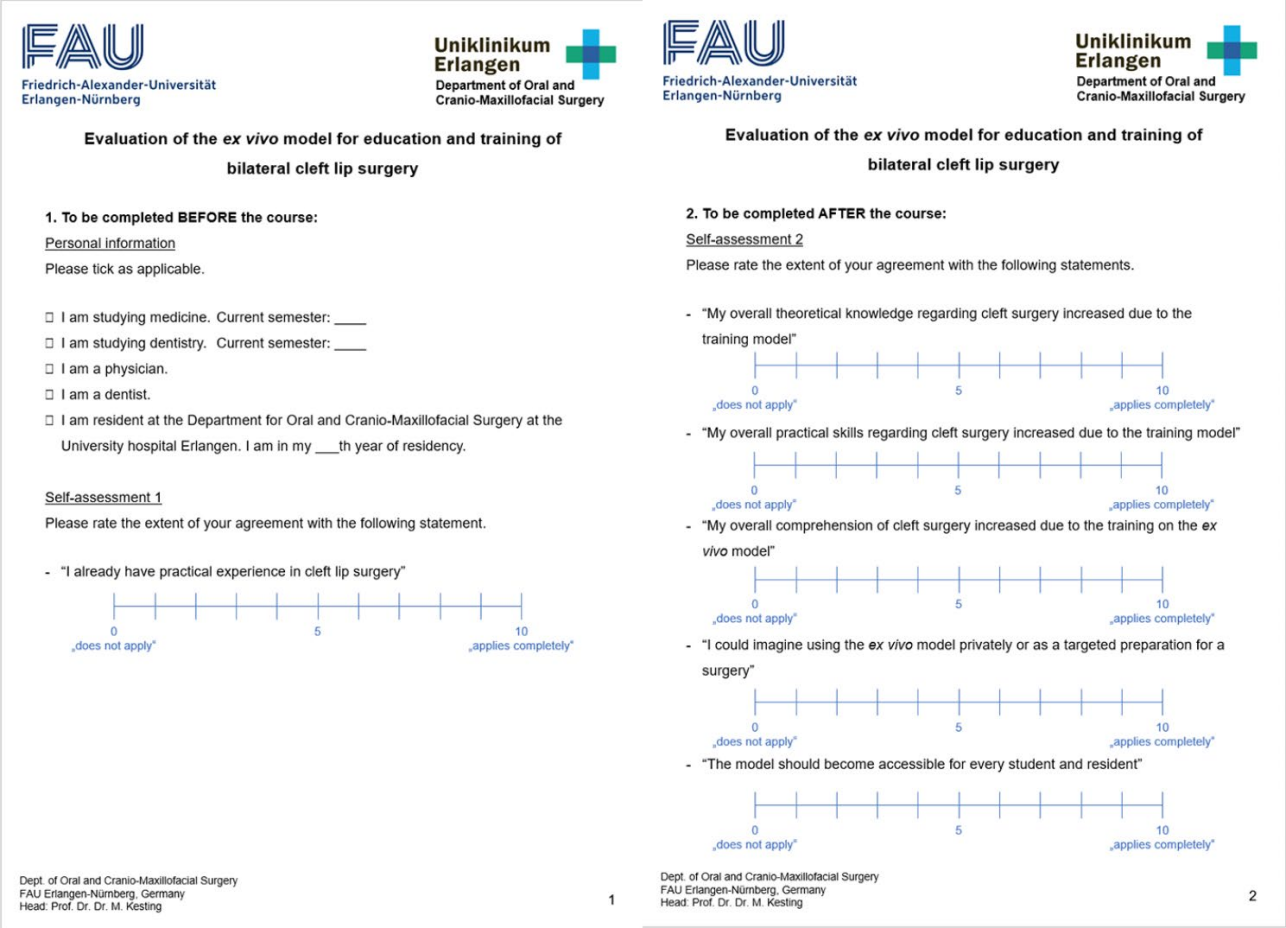
Translated version of the applied, self-designed questionnaire

**Fig. 11:**
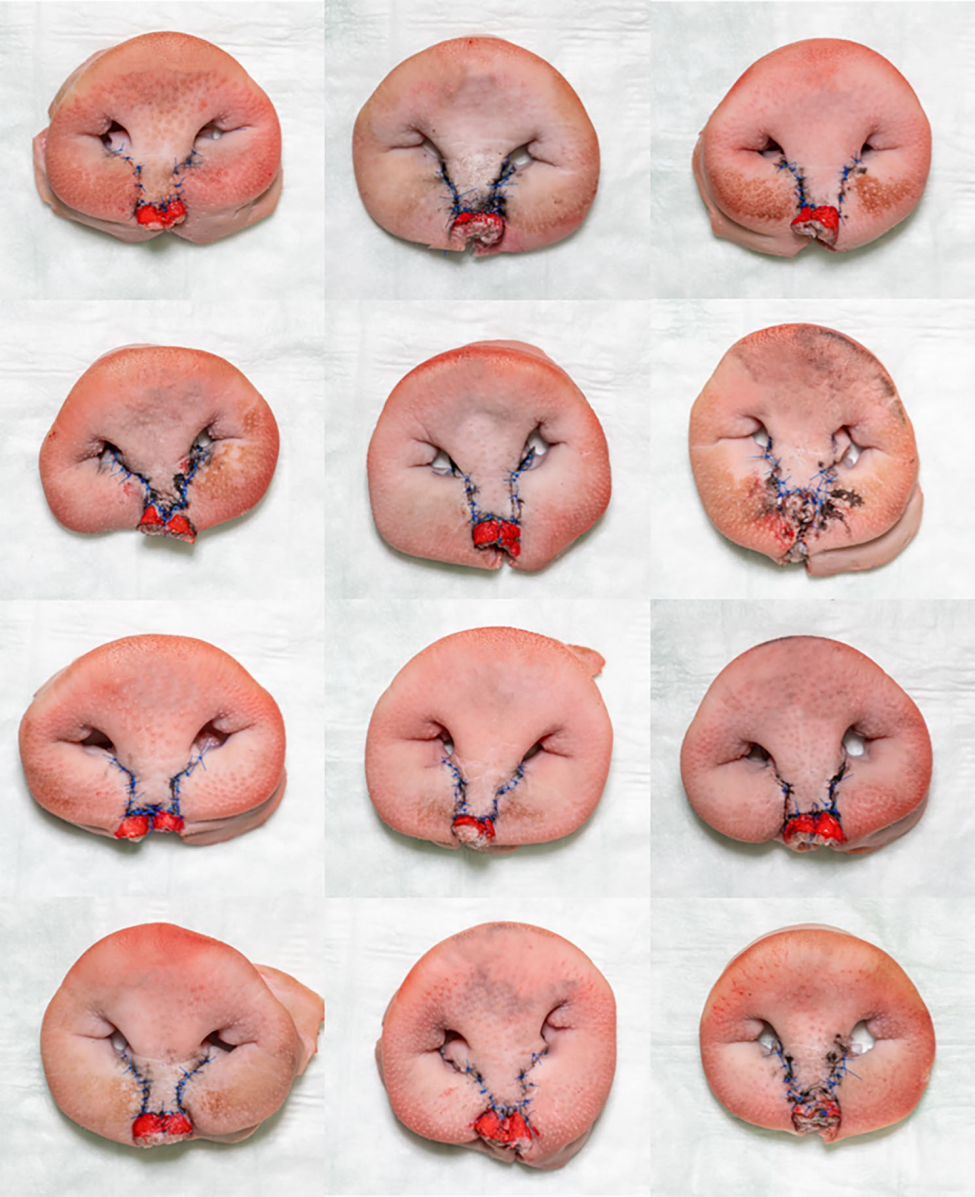
Surgical outcomes after Millard surgery on the *ex vivo* models

After the first successful surgery on the *ex vivo* bilateral cleft model, it was further tested in a course with twelve participants. At the beginning of the course, the participants were handed a German self-assessment questionnaire designed by the authors with continuous answer scales from zero (“does not apply”) to ten (“applies completely”). A translated version of the questionnaire is shown in Fig. 10. Information on the participants’ education levels was gathered and they rated if they “already had practical experience in cleft lip surgery”. Then, the participants were instructed in the creation of the *ex vivo* cleft model and the surgical procedure according to Millard in a lecture. During the course, the surgical steps were again demonstrated on a model until complete closure. The results of the participants’ surgeries were photodocumented (Fig. 11). After the surgical simulation, participants should indicate the level of propriety of the following: “my overall theoretical knowledge regarding cleft surgery increased due to the training model”, “my overall practical skills regarding cleft surgery increased due to the training model”, “my overall comprehension of cleft surgery increased due to the training on the *ex vivo* model”, “I could imagine using the *ex vivo* model privately or as a targeted preparation for a surgery” and “the model should become accessible for every student and resident”.

## Results

By transferring the clinical anatomical conditions of a cleft patient to the porcine snout disc, it was possible to create an *ex vivo* model for bilateral cleft lip surgery. The template could thereby be used several times, as the porcine snout discs were adequately uniform in size. The anatomic features were similar enough to the human situation to permit an easy transfer of the clinical cleft situation.

In the *ex vivo* model, bilateral Millard’s cleft surgery was successfully performed and a high correlation with the clinical surgical procedure of bilateral lip plasty was achieved. Tissue handling reflected the model’s susceptibility, allowing precise execution of the relevant surgical steps of a multi-layered lip construction. However, there were limitations in performing detailed primary rhinoplasty, since the snout disc lacks the three-dimensional structure of the human nose.

All twelve questionnaires of the participants were evaluated. All participants were of the level of a consultant or lower. None of the participants had already performed a bilateral cleft lip surgery on a patient on their own responsibility and all indicated little practical experience (average 1.00, SD 1.76). All participants were able to successfully perform bilateral cleft lip surgery on their *ex vivo* model. The photodocumented outcomes of their surgeries are shown in Fig. 11. The self-assessment after the surgical simulation training indicated a great increase in the participants’ theoretical knowledge (average 8.33, SD 1.61), their practical skills (average 9.42, SD 1.08), and their overall comprehension regarding cleft lip surgery (average 8.92, SD 1.62). Everyone agreed that they might want to use the *ex vivo* model as a private training model or as a preparation for a surgery in the future (average 8.83, SD 1.53). In the participants’ opinion, the *ex vivo* model should become easily available to all trainees (average 8.67, SD 2.35).

## Discussion

The porcine snout disc proved to be a useful *ex vivo* model for creating a bilateral cleft lip situation. By comparing the simulated surgical steps with the clinical case, the model’s good approximation to reality becomes apparent. The relevant surgical steps of the complex procedure of bilateral cleft lip surgery, especially of the lip construction, are implemented. It enables surgeons and trainees to understand the individual surgical steps as well as the procedure itself. Even the process of creating the *ex vivo* model and transferring the landmarks and reference points to the snout disc implicitly deepens the trainee’s understanding of the anatomical situation. In addition, the cadaver model naturally gives a realistic feel to all the different tissue textures that synthetic models cannot yet match [14–16].

There are anatomical and visual differences between the *ex vivo* model and the clinical situation. The porcine snout disc is flat and does not mimic the three-dimensionality of the human nose. Effects of cleft surgery on the columella were therefore less notable than in the clinical case and surgical steps regarding the rhinoplasty are only simulated to a limited extent. However, the narrowing of the nostrils was clearly visible in the *ex vivo *model and even high-fidelity silicone models have not yet been able to provide a satisfactory haptic for rhinoplasty [12]. The *ex vivo* model therefore helps to comprehend the effects of the lip closure on the nasal region, but it does not provide the morphological conditions to perform primary rhinoplasty. Further improvement of this part of the surgical simulation needs to be pursued. Due to its thicker epidermis [18], porcine skin is slightly rougher in its handling than human facial skin. Thus, compared to human skin and models made of silicone, the *ex vivo* model is more tolerant of inaccuracies of the surgeon. This and its double magnification scale decrease the difficulty level of the surgery, which one must be aware of before applying his acquired skills on a clinical case. Yet it is not the aim of the model that every student performs cleft surgery immediately after his surgical simulation training. Depending on one’s purpose, these anatomic differences can also be beneficial. The lower level of difficulty makes the *ex vivo* model highly suited to serve as an education and training model for beginners in cleft surgery. For the students and surgical residents involved in this project, the surgical training on the *ex vivo *model helped them in deepening their understanding of bilateral cleft lip surgery. The qualitative feedback via questionnaires and the personal feedback from the course participants was very positive regarding the possible transfer of knowledge and skills originating from the simulation on the *ex vivo* model. All participants agreed that the model is a valuable training tool and that performing a surgical simulation on the model significantly improves the competencies and skills of residents and colleagues in cleft lip surgery. All advocated the further use of the model to improve training of residents and colleagues in cleft surgery and also showed interest in conducting a similar course for the *ex vivo* model of unilateral cleft lip surgery.

The participants’ positive feedback on the *ex vivo* model and their eagerness to conduct similar surgical courses emphasise the desire and need for learning and training opportunities. When performing cleft surgery on a patient, the surgeon is generally expected to have a high level of expertise, even if it is his first cleft surgery [6]. A surgeon should therefore be able to acquire this very expertise at university hospitals, since one of their main tasks is to provide professional education for medical residents and students [22]. Unfortunately, education and training at universities are hitherto only provided to a very limited extend and the expertise in the field of cleft surgery often seems to lie in the hands of a few specialists. Likewise, haptic high-fidelity models for bilateral cleft surgery are very expensive and hardly available, discouraging interested surgeons and students from deepening their knowledge and enhancing their surgical skills. The mere acquisition of theoretical knowledge is not sufficient, however, as surgical residents are predominantly hands-on learners [23]. The porcine snout disc model intervenes at exactly this problem, since it is both inexpensive and easily available. Using the template technique, the *ex vivo* model is easy to imitate and, due to the minimal financial outlay, may be widely used for the education and training of bilateral cleft lip surgery. It can be easily adapted to personal surgical preferences. This allows practising different incision and surgical techniques as well as simulating different cleft characteristics by creating the corresponding template. The *ex vivo* model offers educational centres an easy opportunity to familiarise their medical residents and students with the field of plastic and reconstructive surgery. A previous study by Bauer et al. [22] found that surgical hands-on courses are a valuable enhancement to the medical curriculum, since they increase the participants knowledge as well as their interest in the corresponding field. According to the authors, providing more practical courses might even lead to more young medical professionals choosing to specialise in surgery.

Besides its suitability to train or educate many residents or students at the same time, the *ex vivo* model is furthermore ideally suited for one’s individual repetitive surgical training. This is an improvement over the high-fidelity silicone models priced about $ 250 per model [24]. The *ex vivo* model is not only of low costs, though, but its availability is almost unlimited, whereas the availability of high-fidelity cleft models was very limited during the COVID-19 pandemic. Moreover, the manufacturing of the model is simple and requires no special equipment. In fact, the information contained in this work enable anyone interested to create an *ex vivo* model themselves, following the maxim by which the plastic surgeon Arthur Barsky had already been teaching [4]: *“Give a man a fish and he will eat for a day. Teach a man to fish and he will for a lifetime”.*

## Conclusion

The *ex vivo* porcine snout disc model described in this study is useful as a simulation model for bilateral cleft surgery and in particular for the surgical steps of the multi-layered lip construction. Unlike high-fidelity models, it is not only inexpensive, but also easily and widely available. It is therefore a valuable tool for education and training. The model allows both surgeons and students to gain experience in cleft surgery, improve their practical skills and to deepen their understanding of the complex surgical procedures. The main limitation is that the model cannot be used to replicate a primary rhinoplasty.

## Data Availability

Most of the data generated or analysed during this study are included in this article. The remaining datasets used and/or analysed during the study are available from the corresponding author on reasonable request.
